# Applicability of Artificial Intelligence-Enabled Chatbots in Medical Physics

**DOI:** 10.7759/cureus.110649

**Published:** 2026-06-11

**Authors:** Avinav Bharati, Deepsekhar Das, Susama R Mandal, Pratik Kumar, Atindra Narayan, Raaj K Bisht, Lalit Takia, Varsha Mishra

**Affiliations:** 1 Medical Physics, All India Institute of Medical Sciences, New Delhi, New Delhi, IND; 2 Ophthalmology, All India Institute of Medical Sciences, New Delhi, New Delhi, IND; 3 Medicine, All India Institute of Medical Sciences, New Delhi, New Delhi, IND; 4 Pediatrics, All India Institute of Medical Sciences, New Delhi, New Delhi, IND

**Keywords:** artificial intelligence (ai), chatbots, chatgpt, deepseek, gemini

## Abstract

Aim

Chatbots are emerging as a new and valuable tool in healthcare, offering a wide range of applications. Their use as a tool in medical physics has immense future potential. This study aimed to evaluate the performance of three artificial intelligence (AI) chatbots - ChatGPT, DeepSeek, and Gemini - in response to questions or queries related to medical physics in oncology.

Materials and methods

A total of 11 questions from the field of medical physics pertaining to oncology were formulated by medical physics experts. These queries were presented to the AI chatbots - ChatGPT 5.2, DeepSeek V 3.2, and Gemini 3.0 - on a predetermined date. Responses were obtained by repeating the same question once for each chatbot. The initial responses were noted and evaluated by three experts based on their correctness, completeness, ease of understanding, reliability, and applicability in the national scenario.

Results

The mean correctness scores were 3.4, 3.81, and 3.09 for ChatGPT, DeepSeek, and Gemini, respectively. Regarding completeness, the DeepSeek gave the maximum responses, that is, 10 complete responses to the 11 questions. No statistically significant difference was foundin real-world applicability score for the three models.

Conclusion

In terms of performance metrics such as correctness, DeepSeek gave better results. None of the chatbots were seen to be good enough to replicate human intelligence in metrics such as correctness, completeness, or real-world applicability. A symbiotic collaboration between AI chatbots and medical professionals is essential for enhancing healthcare delivery.

## Introduction

Artificial intelligence (AI) models with the capability of advanced reasoning and utilizing existing medical knowledge can perform various medical tasks and assist healthcare workers and researchers in performing certain tasks. These models are able to understand various electronic records, such as medical images and videos, and interpret them to give an appropriate response. Chatbots are emerging as a new and valuable tool in healthcare, offering different applications. Their application as a tool in medical physics is hence inevitable. In medical physics, they can be used as a supporting tool for informed decision-making, better patient communication, and streamlining administrative tasks.

ChatGPT, an AI-driven conversational large language model (LLM), has garnered significant scholarly attention since its inception, owing to its manifold applications in the realm of medical science. The healthcare industry is abundant in data and possesses a substantial amount of text, creating an urgent demand for automation. The development of AI enables improved processing and analysis of medical data, unveiling new associations between diseases and treatments. ChatGPT has shown tremendous potential in the medical field, introducing new interaction patterns between doctors and patients in clinical practice, as well as pioneering advancements in healthcare, medical education, and research. A recent study on healthcare workers found that almost one-fifth of them utilized ChatGPT, and this number is increasing with every passing month [1-2].

AI chatbots can be very effective for locations with very scarce healthcare professional services. In such locations, they can augment healthcare facilities by acting as an intelligent tool. If we try implementing AI capabilities with a myopic view of replacing the healthcare professionals, then it will mean working for a lost cause. Therefore, the optimal approach will be to integrate the capabilities of AI chatbots with the expertise and clinical acumen of healthcare professionals rather than replacing human involvement. We must remain steadfast in our ultimate objective of improving healthcare outcomes and quality of care in this digital frontier.

DeepSeek is an AI development firm based in Hangzhou, China. The first version was introduced in November 2023. DeepSeek [3] focuses on developing open-source LLMs. DeepSeek’s DeepThink (R1) is an open-source LLM. Since its introduction, different versions of DeepSeek have been released, but the R1 reasoning model gave a new global recognition to DeepSeek in Jan 2025. The DeepSeek AI assistant - a mobile app that provides a chatbot interface for DeepSeek-R1 - topped the Apple App Store chart, outranking OpenAI's ChatGPT mobile app soon after its launch in Jan 2025.

Despite being capable of offline deployment, DeepSeek presents itself with users’ data privacy concerns such as user data retention and complete developer access to user-generated content without opt-out options. These ethical and regulatory challenges need to be taken care of. However, from the users’ perspective, one of its advantages is its open-source nature [4,5], which enables them to customize the source code as per their requirements. This open source of DeepSeek may promote collaboration between healthcare workers and computer sciences professionals. This unregulated customization may harm its potential in its standardization and reliability in its application in healthcare, research, or any military applications. However, on the brighter side, for localized healthcare applications, it may be customized and fine-tuned for region-specific applications.

Gemini AI is a chatbot developed by Google. The Google DeepMind is the primary team responsible for its research and engineering. Gemini is great at mixing different types of information together. Med-Gemini is in its developmental stage and will be building upon the success of Google’s Gemini models.

The purpose of the present study was to comparatively evaluate the performance of three widely used AI-enabled chatbots - ChatGPT 5.2, DeepSeek V 3.2, and Gemini 3.0 - in responding to domain-specific queries related to medical physics in oncology.

## Materials and methods

Three AI chatbot models, ChatGPT, Gemini, and DeepSeek, were selected for the study. Medical physicists of a tertiary care oncology institute formulated 11 questions (Table 1) to be asked to the chatbots.

**Table 1:** Medical physics question H&N, head and neck; IMRT, intensity-modulated radiation therapy; ITV, internal target volume; PTV, planning target volume; QC, quality control; SBRT, stereotactic body radiation therapy; VMAT, volumetric modulated arc therapy

S. No.	Questions
1	A patient with carcinoma left breast is being planned for radiotherapy post-mastectomy. What modality and what dose is to be planned for delivery?
2	A patient with carcinoma of bilateral breast is being contoured and submitted for radiotherapy planning. What modality must be employed and with what technique to minimize dose to bilateral lung and heart?
3	A patient with carcinoma buccal mucosa is being treated with VMAT technique for radiotherapy. To minimize toxicity to the contralateral parotid (Xerostomia), should we plan with a full or a partial arc?
4	How frequent the patient specific QC of IMRT plans needs to be done/practiced to maintain the accuracy of dose delivery. Do we need to perform it for all the patients before starting the treatment?
5	A case of recurrent H&N carcinoma is being planned for radiotherapy. What are the factors which may alter the planning parameters for dose delivery in H&N re-irradiation?
6	A 55-year-old patient with early-stage left-sided breast carcinoma has undergone breast-conserving surgery. What hypofractionated radiotherapy schedule would you recommend, and what are the dose constraints for the heart and ipsilateral lung?
7	A patient presents with a solitary 2-cm brain metastasis. From a medical physics perspective, what are the planning considerations for stereotactic radiosurgery (SRS) compared to conventional fractionated radiotherapy?
8	A patient with peripheral early-stage non-small cell lung carcinoma is planned for SBRT. What motion management techniques should be employed, and how should ITV and PTV margins be determined?
9	During IMRT planning for carcinoma cervix, what are the recommended dose constraints for organs at risk (rectum, bladder, bowel bag, and femoral heads), and how can plan optimization reduce toxicity?
10	In patients undergoing radiotherapy for locally advanced head and neck carcinoma, what anatomical changes may necessitate adaptive replanning, and what imaging modalities are used to guide adaptive radiotherapy?
11	What periodic quality assurance tests are required for VMAT delivery to ensure dosimetric and mechanical accuracy, and how frequently should they be performed according to international guidelines?

The prompts were prepared on the various dosages and delivery modalities for different forms of carcinoma, namely breast carcinoma and head and neck oncology. Certain prompts catered to queries regarding the bilateral nature of the disease and re-irradiation in a specific scenario. One of the questions included the quality assurance aspect of intensity modulated radiotherapy treatment delivery. All the questions were phrased properly and reviewed by three experts in the field of oncology so that they were not ambiguous and the chatbots could interpret them easily. All the experts were clinical medical physicists with at least 10 years of experience.

The questions were put into the three AI Chatbots, namely ChatGPT 5.2, DeepSeek v3.2, and Gemini 3.0. The answers by each chatbots were copied and pasted in Microsoft Word (Microsoft Corp., Redmond, WA, USA). The answers provided by the chatbots were analyzed by three experienced medical physicists independently.

A tabulated proforma was designed to score the results based on the specific parameters such as correctness (completely wrong / wrong / partially correct / absolutely correct), completeness (incomplete/ partially complete / complete), language and readability, irrelevant data (none, <25%, 25-50%, >50%), real-world applicability of the advice (word to word) in Indian scenario (none / partially / completely), and grade of reliability (invalid / not reliable /moderately reliable / fairly reliable / highly reliable). The responses on repeating the question were also evaluated to determine the repeatability of the chatbot responses. Statistical analysis was performed by ANOVA one-way samples using the Origin software (OriginLab Corporation, Northampton, MA, USA).

The experts’ judgements were aligned with internationally recognized standards, including recommendations from the European Society for Radiotherapy and Oncology (ESTRO), the American Association of Physicists in Medicine (AAPM), and the International Commission on Radiation Units and Measurements (ICRU). Dose prescription principles were aligned with ICRU Reports 83, 62, and 50. IMRT quality assurance, treatment planning, and patient-specific verification practices were evaluated in accordance with relevant AAPM Task Group reports (e.g., TG-218, TG-142, TG-119). Contouring and target delineation considerations were assessed using ESTRO consensus guidelines, ensuring alignment with contemporary global best practices in radiotherapy physics.

## Results

The total mean sentences were 27.63 ± 9.17, 44.72 ± 9.62, and 27.18 ± 6.72 for ChatGPT, DeepSeek, and Gemini, respectively (Tables 2-4). Statistically significant difference was found in response length between ChatGPT and DeepSeek. Similarly, difference was observed between DeepSeek and Gemini, with statistical significance. DeepSeek scored the highest mean among all the three models. The total mean words used were 388.72 ± 108.09, 490.81 ± 111.26, and 493.09 ± 39.31 for ChatGPT, DeepSeek, and Gemini, respectively. A statistically significant difference was found between ChatGPT versus DeepSeek and ChatGPT versus Gemini. ChatGPT scored the highest total mean words among the three chatbots. Mean reliability scores were 4 ± 0.63, 3.63 ± 0.67, and 3.54 ± 0.68 for ChatGPT, DeepSeek, and Gemini, respectively. No statistically significant difference was found between ChatGPT, DeepSeek, and Gemini.

**Table 2:** Scoring of answers by AI model: ChatGPT Note: the terms A1, A2... refer to answers to questions 1, 2… AI, artificial intelligence

	A1	A2	A 3	A 4	A 5	A6	A7	A8	A9	A10	A11
Correctness (1-4)	4	4	3	3	3	3	4	3	4	3	4
Completeness (incomplete/complete)	Incomplete	Complete	Complete	Complete	Complete	Complete	Complete	Incomplete	Complete	Complete	Complete
Language and readability											
Ease (Flesch Kincaid score)	31.83	42.04	35.65	36.48	46.11	34.72	40.42	38.44	41.04	35.39	42.56
Total sentences	24	22	17	24	45	31	28	24	19	26	44
Total words	477	410	220	267	487	496	440	320	254	525	380
Irrelevant data (NA / 1-25% / 25-50% / >50%) (percentage)	NA	1-25%	NA	1-25%	NA	NA	NA	NA	1-25%	1-25%	NA
Additional relevant data (no/some)	Some	Some	No	Some	No	Some	No	Some	Some	Some	No
Real-world applicability (word to word) in the Indian scenario (none/partially/entirely)	Entirely	Partially	Entirely	Partially	Partially	Entirely	Partially	partially	Partially	Partially	Entirely
Grade of reliability	5	3	4	4	4	4	4	4	3	4	5
Comments	The platform prompts the user to ask further question. For first question, the Platform concludes that the final decision should be based on the tumor characteristics. In some queries, chatbot was taking a bit longer. Responses A9 and A11 took a bit longer, and the chatbot took 1 minute 6 seconds and 1 minute 28 seconds to respond, respectively. The time was mentioned in the response screen.										
Response on repeating same questions twice (no change/added information/changed information)	Added information	Added information	No change	No change	Added information	No change	No change	No change	No change	No change	Added information

**Table 3:** Scoring of answers by AI model: DeepSeek AI, artificial intelligence

	A1	A 2	A3	A4	A5	A6	A7	A8	A9	A10	A11
Correctness (1-4)	3	4	4	4	4	4	4	4	3	4	4
Completeness(incomplete/complete)	Complete	Complete	Complete	Complete	Complete	Complete	Complete	Complete	Complete	Complete	Incomplete
Language and readability											
Ease (Flesch Kincaid score)	28.18	49.78	41.41	43.56	27.19	32.19	46.32	35.78	45.65	38.44	29.66
Total sentences	31	42	39	52	62	50	35	46	35	44	56
Total words	228	560	485	546	630	540	490	510	440	380	590
Irrelevant data (NA / 1-25% / 25-50% / >50%) (percentage)	NA	NA	NA	25-50%	1-25	NA	NA	NA	NA	NA	1-25%
Additional relevant data (no/some)	Some	No	No	No	Some	No	No	No	Some	No	No
Real-world applicability (word to word) in the Indian scenario (none/partially/entirely)	Entirely	Partially	Entirely	Partially	Partially	Partially	Partially	Entirely	Partially	Partially	Partially
Grade of reliability	4	3	5	3	4	3	3	4	3	4	4
Comments	The platform gave a summary of its response. For question 5, the users had to seek response from the platform 4 times as the server was busy. Summary at the end.										
Response on repeating same questions twice	No change. For question 4, the response took time as the server was busy.										

**Table 4:** Scoring of answers by AI model: Gemini AI, artificial intelligence

	A 1	A 2	A3	A4	A 5	A6	A7	A8	A9	A10	A11
Correctness (1-4)	3	3	3	3	3	3	3	3	3	4	3
Completeness(incomplete/complete)	Incomplete	Incomplete	Complete	Incomplete	Complete	Complete	Incomplete	Complete	Complete	Incomplete	Complete
Language and readability											
Ease (Flesch Kincaid score)	31.83	35.20	31.79	27.85	24.03	40.36	30.29	25.74	28.72	37.22	22.84
Total sentences	24	24	25	20	36	32	21	32	40	21	24
Total words	477	488	518	437	558	522	463	470	523	477	455
Irrelevant data (NA / 1-25% / 25-50% / >50%) (percentage)	NA	25-50%	1-25%	25-50%	25-50%	25-50%	25-50%	25-50%	25-50%	1-25%	NA
Additional relevant data (No/some)	Some	Some	No	No	No	No	No	Some	Some	No	No
Real-world applicability (word to word) in Indian Scenario (None/Partially/Entirely)	Entirely	Partially	Entirely	Partially	Partially	Partially	Entirely	Partially	Partially	Partially	Entirely
Grade of reliability	4	2	4	4	4	3	3	4	4	4	3
Comments	Suggestion to consult an expert at the end as well as at the beginning.										
Response on repeating same questions twice	No change	Reduced information	Reduced information	Reduced information	No change	Reduced information	Reduced information	No change	No change	No change	Reduced information

Mean correctness scores were 3.4 ± 0.52, 3.81 ± 0.40, and 3.09 ± 0.30 for ChatGPT, DeepSeek, and Gemini, respectively. A significant difference was found between DeepSeek and Gemini statistically. DeepSeek had the highest mean correctness among all the three models. The completeness of response was found to be more in the case of DeepSeek answers. The response was complete for 9, 10, and 6 queries to ChatGPT, DeepSeek, and Gemini, respectively. Mean ease-of-readability scores were 38.6 ± 4.2, 38.01 ± 7.94, and 30.53 ± 5.48 for ChatGPT, DeepSeek, and Gemini, respectively. DeepSeek showed comparatively more variation (standard deviation 7.94), indicating slightly higher inconsistencies in sentence complexity. A statistically significant difference was found between ChatGPT versus DeepSeek and ChatGPT versus Gemini. ChatGPT response was comparatively easier to understand in comparison to the other two chatbots.

In case of mean irrelevant data values, a statistically significant difference was found between ChatGPT and Gemini only. Gemini scored the highest mean among all three model for irrelevant data. No statistically significant difference was found between ChatGPT, DeepSeek, and Gemini for mean additional relevant data metrics. ChatGPT scored the highest mean among all three chatbots. Similarly, no statistically significant difference was found between ChatGPT, DeepSeek, and Gemini in real-world applicability index or score.

**Figure 1:**
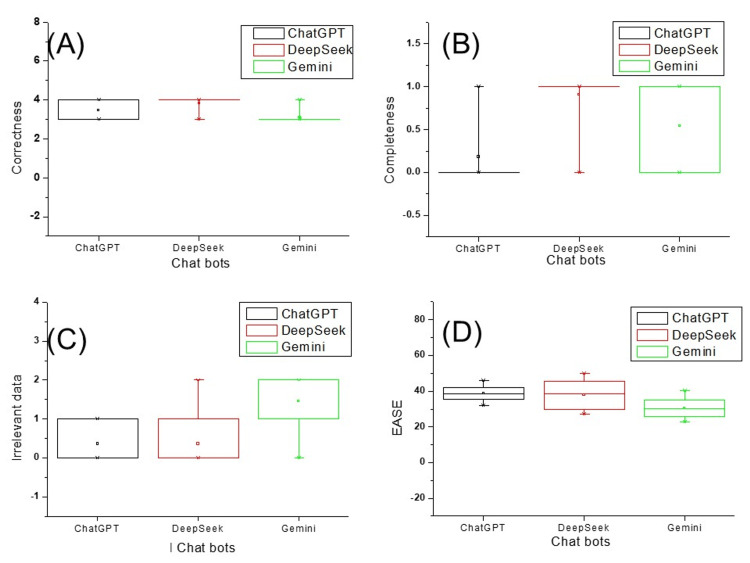
Statistical comparison between chatbots for metrics such as correctness, completeness, irrelevant data generated, and ease of readability The figure shows a comparison of ChatGPT, DeepSeek, and Gemini across four qualitative and quantitative performance categories using box-and-whisker plots. (A) Correctness: a statistically significant difference was observed between DeepSeek and Gemini. (B) Completeness: a statistically significant difference was found between ChatGPT and DeepSeek. (C) Irrelevant data: a statistically significant difference was found between ChatGPT and Gemini. (D) Ease: a statistically significant differences were noted between ChatGPT and DeepSeek, as well as between ChatGPT and Gemini.

**Figure 2:**
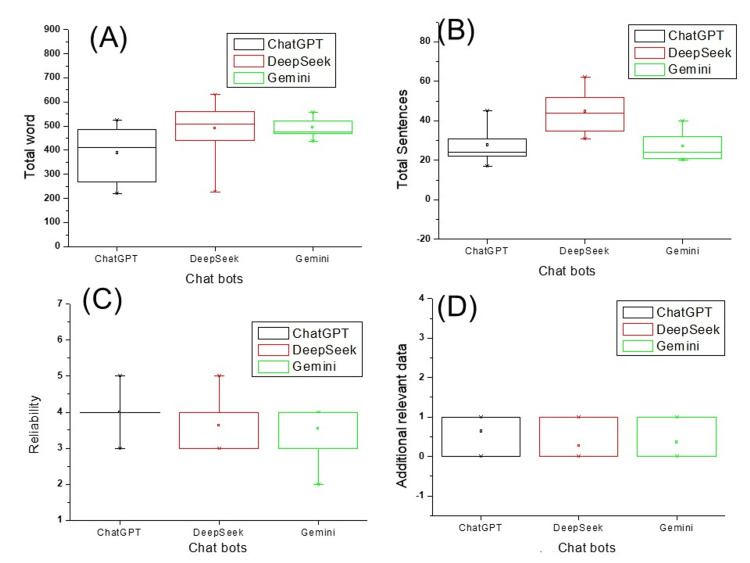
Statistical comparison between total words, total sentences, reliability, and additional relevant data This figure presents quantitative and reliability metrics for the three AI models. (A) Total words: statistically significant differences were found between ChatGPT versus the other two models. (B) Total sentences: statistically significant differences were found between DeepSeek and the other two models. (C) Reliability (grade of reliability): no statistically significant difference was found. (D) Additional relevant data: no statistically significant differences observed.

Statistical analysis was performed by ANOVA one-way samples using Origin software. Interclass correlation (ICC) analysis was performed for ease, total sentences, and total word data for inter-rater agreement among three raters who were qualified medical physicists. The average ICC was found to be 0.98 for all the three models (ChatGPT, DeepSeek, and Gemini). Kappa coefficient was analyzed for correctness, completeness, grade of reliability, irrelevant data, additional relevant data, and real-world applicability for all three models. The average Kappa coefficient was found to be 0.82, indicating strong agreement among the raters. Figures 1, 2 show the statistical comparison between the three chatbots’ response.

## Discussion

A typical medical chatbots involves four main modules: understanding text, management of dialog, database layer, and text generation. The most common technique for text understanding and dialog management is pattern matching [6]. AI-based chatbots can be used for emergency as well as regular health consultation for immediate and personalized health needs. However, the ethical constraints must be considered before making it or accepting it as a regular clinical tool. Also, the human intervention and verification is very crucial before implementation.

AI models using LLM responds to the prompt after understanding. An instantaneous response is obtained after the model analyses the text based on certain statistical relationship between different words. A phenomenon called hallucination [7] refers to a pitfall in the use of AI-driven technology. Hallucination leads to fabrication or citing of inaccurate references, which ultimately generates nonsensical or inaccurate content.

The medico-legal and ethical considerations are also one aspect of use of AI chatbots. The government must take required steps to safeguard individuals’ interest if they really want to promote AI chatbots in any field. Important concerns associated with increased use of AI chatbots are data security, transparency, and privacy. For a wide acceptability of chatbots, an efficient regulatory mechanism needs to be followed along with the technological advancements. National data protection laws and data encryption protocols must be implemented to mitigate such apprehensions associated with AI chatbots. India did not have a standalone law or framework to govern data protection until 2023. The Information Technology Act 2000 and rules notified thereunder formed the basis around which the data protection framework revolved. In August 2023, the government of India implemented the Digital Personal Data Protection Act 2022 (DPDP Act), which formed the personal data protection and regulatory regime in India. The privacy policy of the DeepSeek is very disturbing. The users are compelled to accept them without any optout option. This policy provides the company with the right to use the collected data to improve its product. ChatGPT, however, does not store user data, and it is never used for model training.

In the current study, we found that the DeepSeek chatbot generated responses with the highest correctness score, whereas DeepSeek outperformed all the other chatbots in terms of completeness of the responses provided. A higher Flesch-Kincaid Reading Ease score [8] indicated the ease to read and understand or requirement of a lower level of understanding. ChatGPT and DeepSeek responses had almost similar ease scores. The responses generated by ChatGPT were the shortest in terms of number of words utilized in response to the question posed to them. The number of sentences used by ChatGPT was almost equal to as in the case of Gemini. The responses were also comprising of some or no irrelevant data generated by chatbots. Irrelevant data were generated in four, three, and nine responses of ChatGPT, DeepSeek, and Gemini, respectively.

The response of a chatbot has different lengths due to difference in complexity of the query presented to the chatbots. Also, the chatbot may be rule-based or AI-based [9] in terms of its design. Rule-based chatbots follow predefined rules and logic, while AI chatbots leverage machine learning and natural language processing to understand and respond to user input more naturally. Although all our chatbots were AI-enabled, the training data used in the design and training of the chatbot also determines how precise and detailed the explanations generated by Chatbots are. Word count of the response of the chatbot also depends on its design, such as whether it summarizes previous messages or deletes older ones to stay within token limits [10]. Additional relevant data were generated in six, three, and four responses for ChatGPT, DeepSeek, and Gemini, respectively [11]. It needs to be mentioned that the ChatGPT Platform prompted the user to ask further question every time it concluded its response. DeepSeek summarized its response every time it finished its response, while Gemini concluded with a suggestion to consult an expert at the end as well as at the beginning of its response. On repeating the same question, the chatbots responded slightly differently. ChatGPT provided some new information at least four times. While the DeepSeek showcased no change in its response upon repeating the question, the Gemini AI chatbot responded with lesser or reduced information at least six times in comparison to the first time [12-13].

By systematically analyzing expert-formulated radiotherapy and quality assurance-based questions, the study intended to determine whether AI chatbots can function as reliable decision-support tools in medical physics practice. The broader objective was to explore their potential role as adjuncts to human expertise rather than replacements, thereby examining the feasibility of integrating AI-driven conversational systems into oncology-focused medical physics workflows.

The role of medical professionals in managing complex medical problems and maintaining patient trust is an irreplaceable part of healthcare [14-15]. A medical or healthcare professional’s role is limited to not only diagnosing and treating diseases but also building rapport with trust and empathy, which requires years of experience and knowledge in healthcare and critical thinking, something that chatbots are incapable of performing [16-17].

AI chatbots can perform tasks such as patient triaging, psychological health counselling, initial consultation [18] to understand the symptoms, and scheduling appointments. In addition to these, they are also a very important tool in healthcare research [19] for performing tasks such as literature review, summarizing research, and writing research articles and proposals.

## Conclusions

In our study, the responses were generated from three chatbots. It was found that correctness of responses for DeepSeek was better than that of Gemini and ChatGPT. In readability scores, i.e., ease of understanding, ChatGPT and DeepSeek responses were found to be of similar standards. However, none of the chatbots were found to be sufficiently capable of replicating human intelligence across the evaluated performance metrics, including correctness, completeness, real-world applicability, and ease of language.

Technological breakthroughs, patient and provider acceptance, ethical and legal resolutions, and improved regulatory frameworks will decide how the mankind accepts these AI chatbots. Therefore, if we try to implement the AI independently or as standalone system to replace a human healthcare professional, then it may fail entirely. The limitations of regional settings too have a very important aspect if we want to use chatbots independently. The correct approach is integrating AI-enabled chatbots with humans’ intelligence.
